# In-Process Orbiting Laser-Assisted Technique for the Surface Finish in Material Extrusion-Based 3D Printing

**DOI:** 10.3390/polym15092221

**Published:** 2023-05-08

**Authors:** Pu Han, Sihan Zhang, Zhong Yang, M. Faisal Riyad, Dan O. Popa, Keng Hsu

**Affiliations:** 1 Ira A Fulton Schools of Engineering, Arizona State University, Tempe, AZ 85212, USA; 2 J. B. Speed School of Engineering, University of Louisville, Louisville, KY 40292, USA

**Keywords:** material extrusion-based 3D printing, surface roughness, surface finish, surface heating, laser process

## Abstract

Material extrusion-based polymer 3D printing, one of the most commonly used additive manufacturing processes for thermoplastics and composites, has drawn extensive attention due to its capability and cost effectiveness. However, the low surface finish quality of the printed parts remains a drawback due to the nature of stacking successive layers along one direction and the nature of rastering of the extruded tracks of material. In this work, an in-process thermal radiation-assisted, surface reflow method is demonstrated that significantly improves the surface finish of the sidewalls of printed parts. It is observed that the surface finish of the printed part is drastically improved for both flat and curved surfaces. The effect of surface reflow on roughness reduction was characterized using optical profilometry and scanning electron microscopy (SEM), while the local heated spot temperature was quantified using a thermal camera.

## 1. Introduction

Additive manufacturing (AM) refers to the use of a computer-aided design (CAD) model in which the required materials are deposited layer by layer to produce three-dimensional (3D) items [1,2]. By virtue of its accessibility and productivity, additive manufacturing based on material extrusion has become a prevailing process for the fabrication of three-dimensional thermoplastic composites and polymers [3,4,5]. The process of 3D printing polymer objects using additive manufacturing is based on material extrusion. In this process, a heated nozzle is adopted to shape a thermal plastic filament. Meanwhile, the position of the nozzle is controlled by a motion system to ensure its movement along the predetermined path. The space rendered by the cross-sectional boundaries of the item to be fabricated at each layer can be filled by the extruded material following along the path. Typically, the temperature of the nozzle should be kept below the melting temperature of the feedstock material and above its glass transition temperature, so that the melted polymer can flow. In this way, the printing process fabricates a 3D item through multi-layer deposition [6,7,8]. The 3D printing process starts from path planning and slicing, whereby the temperature is controlled, the motion command is generated, and the coordinates are determined. Subsequently, the printing job is performed at proper temperatures with material extrusion and well-designed nozzle movements as a response to the temperature control and motion instruction. Thus, it is possible to control the quality of the item printed and its fabrication time [9,10]. Additive manufacturing based on material extrusion is widely applied in many sectors, such as aerospace [3,11], biomedicine [12,13], microfluidics [14], and electric sensors [15]. Various materials can also be used for the building of three-dimensional items [16,17]. Nonetheless, this technique leads to poor surface roughness of prints, which limits its applications. 

The extrusion-based fabrication process itself gives rise to poor surface smoothness because of the multi-layer deposition of the material, with a thickness of 0.1 or 0.2 mm for each layer [18]. The nozzle, due to its shape, determines the roundness of the material extruded. Due to the low level of surface reflow, the side surface is dominated by this round shape, leading to high surface roughness of the 3D item printed. Hence, repeated wave shapes can be perceived from the side surface.

Great efforts have been made to explore how items fabricated through material extrusion-based 3D printing can embrace improvement in their surface finish. These attempts at improving surface smoothness and geometrical accuracy are mostly concentrated on the identification of optimal printing parameters [19,20,21,22,23,24,25]. The surface roughness of printed objects has been calculated through mathematical models of the construction with related parameters [26,27,28,29,30]. The surface finish is also improved with a hot cutter or other post-processing techniques in another approach [27] and with CNC milling [31]. Despite their promoting effects on the surface finish, these techniques are faced with some limitations arising from the sample size. Dimethyl ketone solution finishing has also been investigated as a post-processing chemical technique [32,33]. Meanwhile, attention is also being paid to chemical vapor treatment [34]. In the pursuit of improvement in the surface finish, the technique first utilized on metals is post-processing laser treatment [35,36,37], which was recently employed in the fabrication of printed polymer items for surface finish improvement [38,39,40]. Nevertheless, these solutions all require a higher post-processing investment in the application of a post-processing technique or fail to thoroughly address the issue. Work on addressing the surface roughness issue of 3D-printed parts with in-process techniques has never been reported.

In this work, an in-process local heating approach based on the use of an orbiting laser in the material extrusion-based process is presented. The side surface with various wave shapes is heated with a laser to above the melting or glass transition temperature to ensure that it is capable of reflowing. Such side surface has a higher surface energy than other, smoother surfaces as a result of its wave-shaped characteristic. Surface tension plays a driving role in reducing the surface energy, because it can give the molten polymer a higher surface-reflowing capacity to smooth the side surface. For the purpose of preventing small exterior structures from being reduced, merely a shallow region in a small size is heated up with the laser. This local heating approach enhances surface reflowing and improves the surface finish through filling each extrusion-triggered gap between the layers as well as any other uneven features. This heating approach is applied in the process of printing to optimize the surface finish. In addition to the characterization of surface roughness, efforts are also made in this work to discuss how mechanical strength and fracture behavior as well as chemical structure are affected by laser surface heating.

## 2. Materials and Methods

### 2.1. Orbiting Laser Surface Heating Apparatus

**Figure 1:**
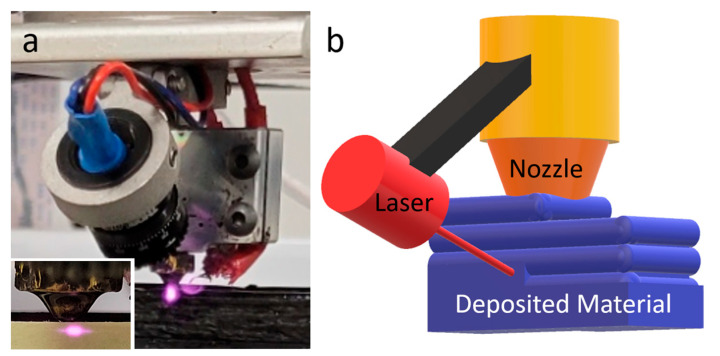
(**a**) Orbiting laser heating apparatus; (**b**) schematic diagram of the heating process.

A commercial 3D printer (Type A Machine Series 1, San Francisco, CA, USA) was used herein to build the heating apparatus. There are a laser source and a heat block in the customized orbiting laser print head (Figure 1a). The orbiting laser source is controlled using a bevel gear set, which is driven by a stepper motor, so that 360° rotation is possible. The source for the laser heating comes from the application of the 808 nm diode module. Partial visibility for safety and small size for operation are the two reasons for this choice of the source. 

To correctly determine the location of the laser source, additional movement controlling commands are generated from the postprocessor created through an algorithm (Figure 1b). There are immediate pauses in printing when the laser source is orbited under an additional movement command (three-axis movement, extrusion). To ensure that 360° rotation consumed less than 1 s, a high value was set for the orbiting speed, so that the pause could be less prone to laser burning. During a specified movement, the laser applied in this method rotates to ensure the correctness of its position before making any change in the direction of the nozzle movement. Located 0.6 mm beneath the nozzle is the major z-position for the rectangular focal point (1 × 0.6 mm) of the laser (Figure 1a). To be specific, considering the 0.2 mm height of the layer, the laser is targeted primarily at the parts below the nozzle, especially the three layers beneath, to avoid heating the current molten layer (which can be adjusted in the case of overhanging features). Hence, it is possible to simultaneously heat two-layer boundaries. Therefore, there is no need to make any post-processing thermal treatments because surface heating occurs in the process of printing, whereby surface reflow is induced.

### 2.2. Temperature Gradient

**Figure 2:**
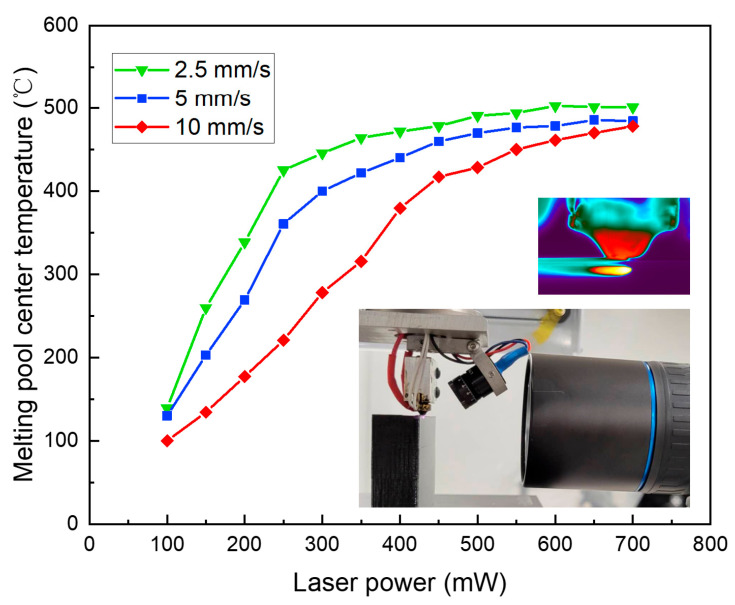
Temperature profile of the heated region at different laser powers and printing speeds.

A thermal camera (FLIR a6753sc, Wilsonville, OR, USA) was used herein to measure the in-process changes in the temperature of the heated parts. In this work, the camera was put ahead of the laser-treated surface in a horizontal direction. Different printing speeds and laser powers lead to changes in the heated size. As for the temperature gradient, its reference is determined to be the maximum point of the thermal profile in the heated region (Figure 2). As shown in Figure 2, the laser power of 700 mW and the printing speed of 5 mm/s are the parameters for the thermal image. According to the surface temperature, even when the laser power is merely 200 mW, on-surface polymer material degrades at the printing speed of 2.5 mm/s. However, visible degradation was not observed on the samples, which could possibly be due to the thin depth of degradation or the quantity of degraded material. This technique was further investigated by examining the influence and depth of degradation. In addition, the acceptability of such degradation for the pursuit of improvement in the surface finish was also identified.

### 2.3. Sample Preparation

**Figure 3:**
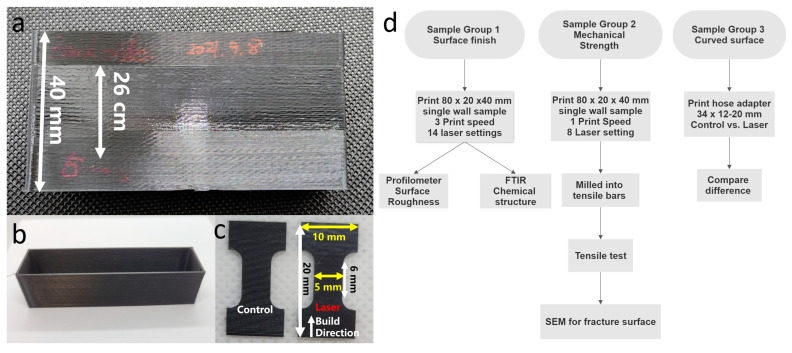
(**a**) Printed rectangular box sample for surface roughness; (**b**) printed rectangular box without top and bottom for tensile test; (**c**) milled tensile bars; (**d**) flow chart of the three groups of samples.

The aforementioned heating apparatus was employed to fabricate all prints herein, for which polylactic acid (PLA) material (MakerGear black PLA, Beachwood, OH, USA) was used as the filament. The 808 nm laser is allowed to have higher absorption with the use of this black filament. All printed items were created with an E3D brass nozzle that is 0.8 mm in size. The layer thickness of the track for deposition was 0.2 mm. The nozzle was maintained at 195 °C, while the building plate was kept at 60 °C. Thirteen laser power settings, with an interval of 50 mW from 100 to 700 mW, and three printing speeds, 2.5, 5, and 10 mm/s, were investigated in this work. A laser power meter (Thorlabs, Newton, MA, USA) was used to measure the laser power output. The entire range of power yielded by the laser diode is fully covered in the laser power range herein. As observed, the diode would be totally damaged if the power were higher than 800 mW. The samples created herein were divided into three groups. The preparation flow chart of the relevant samples is shown in Figure 3d.

The first sample group is for surface roughness. The three samples used for the measurement of surface roughness were produced at varying printing speeds. They are all rectangular boxes that have neither a top nor bottom wall, in a size of 80 mm × 20 mm × 40 mm (L, W, H). The slicing software was applied to slice each box into 200 layers, with each layer being 0.2 mm high. In each laser setting, 10 layers were utilized to constitute a height of 2 mm. For the 13 laser settings introduced above, the central area is dominated by the 26 mm laser-operated region (Figure 3a). The 8 mm top and 6 mm bottom were not operated with the laser, to leave the control regions with a height of 14 mm. Inconsistent extrusion-trigged incorrect data is not included in this work.

The second sample group is for mechanical strength. At 5 mm/s, the printed samples for the tensile test were fabricated under the laser power with an interval of 100 mW, from 0 to 700 mW. As shown in Figure 3b, there is neither a top nor bottom wall in the rectangular box originally printed. Five samples (Figure 3b) were milled with a Bantam tools milling machine (Bantam tools, Peekskill, NY, USA) from the front wall, in which melting during milling was avoided by water cooling. As shown in Figure 3c, the tensile bar in this work is 10 mm × 20 mm in size [41,42]. Due to the focus on the exterior surface, no design of a standardized tensile bar was utilized herein. Laser heating was operated to treat the entire height in each laser sample. Hence, laser heating had some effect on the fracture surface. Meanwhile, whether there is an interlayer interface for the fracture surface can be judged by the layer boundaries on the other side without laser heating.

The third sample group is for curved surfaces. With regard to curved surfaces, whether the heating technique is capable was demonstrated through the designing and printing of a customized hose adapter, which is 34 mm in height, 12 mm in top diameter, and 20 mm in bottom diameter. The moving direction of the nozzle was varied at a high rate to test the capability of this technique on the fabrication of curved surfaces.

### 2.4. Surface Roughness

The sample surface was characterized using a profilometer (Dektak 8M, Veeco, Plainview, NY, USA) with a scanning period of 80 s, a scanning length of 34 mm, a force of 3 mg, and a resolution of 1.417 μm.

### 2.5. Mechanical Testing

The tensile bars milled were tested using a tensile testing machine (MTI-2K, Measurement Technology Inc., Marietta, GA, USA). Among the five samples in each group, tensile testing was conducted on four prints, with one sample acting as a substitute in the case of testing failure or abnormal data. Tensile testing was carried out at a displacement speed of 5 mm/min and a pre-load of 30 N.

### 2.6. Fourier Transform Infrared Spectroscopy (FTIR)

FTIR (Perkin Elmer Frontier, Waltham, MA, USA) was performed on four samples (control, 500 mW at 5 mm/s, 700 mW at 5 mm/s, and 700 mW at 2.5 mm/s) to further investigate the effect of the laser on the chemical structure of the PLA at the surface. The wavenumber used was from 5000 cm^−1^ to 400 cm^−1^. Diamond attenuated total reflection (ATR) mode was used, and 100 scans were performed for each sample. Due to the high surface roughness of the control sample (with a wavy surface feature), while the ATR only measures the contacted area, a printed bulk material was used as the control sample to avoid errors from the surface roughness difference.

## 3. Results and Discussion

### 3.1. Surface Roughness

**Figure 4:**
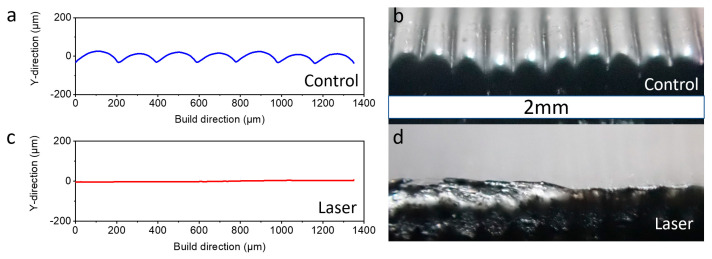
Profilometry data of control (**a**) and 2.5 mm/s 700 mW laser (**c**) sample; optical image of control (**b**) and 2.5 mm/s 700 mW laser (**d**) sample from the side (same scale bar).

The laser-treated sample processed at 2.5 mm/s under the setting of 700 mW and the control sample were compared with regard to their surface morphology (Figure 4a,c). The surface regions (same parameters, not the same location) were also observed through optical imaging (Figure 4b,d). The nearly flat surface in the laser-treated sample, in comparison with the height of roughly 58 μm in its original wave shape, shows that the surface finish has been improved significantly.

**Figure 5:**
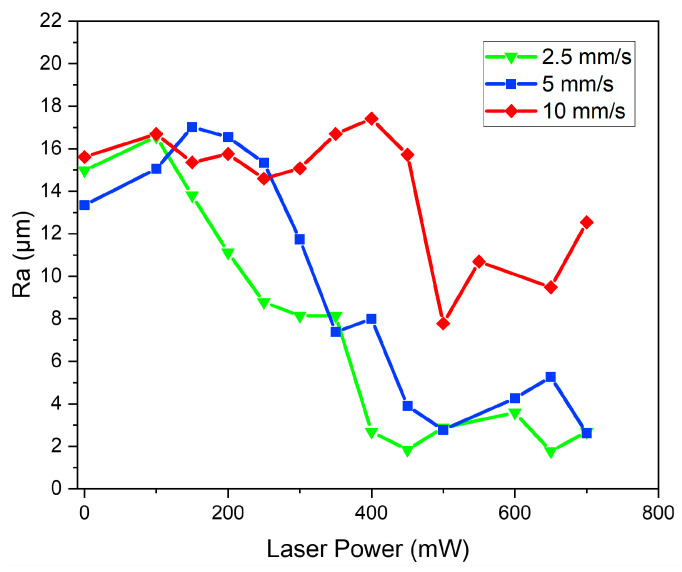
Surface roughness of samples with different laser powers and print speeds.

Figure 5 shows the values of roughness (Ra) obtained from the 14 settings of laser power (with an additional setting for the control sample at 0 mW) at the aforementioned three speeds. For each printing speed setting, the control sample was found to have around 15 μm for its Ra.

The laser reaching 450 mW was not found to have a significant impact on the Ra of the samples treated at 10 mm/s (Figure 5). Accordingly, 450 mW is the starting point for visible surface heating. When the laser power increases to a level as high as 700 mW, there is improvement in the surface heating feature. From visual observation, there is somewhat better surface smoothness and a significant enhancement in light reflection in the samples treated at 10 mm/s compared with the control sample. The surface curvature cannot be covered and fully reflowed by the laser due to its low density at high printing speeds (Figure 4a). However, it can polish the deposited track’s side. Noticeably, there is no obvious improvement in reflection at 10 mm/s when the laser power is lower than 400 mW. At 10 mm/s, the heated region rises to a temperature of 380 °C almost linearly with the increase in laser power to 400 mW. Subsequently, there is a lower slope in the increase in its temperature after 450 mW, as shown in Figure 2, hinting at the improvement of its steadiness at 417 °C. It can be inferred that the surface heating at 10 mm/s, for enough surface reflow, requires a central region temperature of 417 °C.

In Figure 5, the Ra of the samples treated with laser heating at 5 mm/s is represented by the red circle line. From 0 to 250 mW, there is no significant change in the value of Ra. At 400 °C (Figure 2) with the laser power of 300 mW, the reflection is found to have a visible change. When the laser power increases, there is a decline in the value of Ra (Figure 5). At 460 °C (Figure 2) with the laser power higher than 450 mW, the surface roughness is observed to have remarkable improvement. Noticeably, in comparison with the value at 10 mm/s, the region heated, as a result of the lower printing speed, allows more time at the higher temperature. Hence, the Ra increases significantly despite the 461 °C temperature of the heated region at 10 mm/s when the laser power is 600 mW. In addition, power divided by printing speed is used as the laser power input. This suggests the same input at 5 mm/s when the laser power is 300 mW and at 10 mm/s when the laser power is 600 mW.

Herein, 2.5 mm/s is the minimum of the printing speeds. Within 150–400 mW, the Ra is found to decline. As shown in Figure 5, with slight fluctuations, it gains steadiness at roughly 2 μm. At 200 mW (338 °C), the first point of the polishing effect can be observed (Figure 2). From 400 to 700 mW, similar surface features are visible, while there is a change in reflection behavior.

Based on the results above, the orbiting laser-assisted technology showed significant improvement on the surface finish of the 3D-printed parts. Therefore, by locally heating the side surface to above its melting temperature, a sufficient amount of material at the surface reflows and fills the uneven features that are created due to the extrusion-based deposition process. This local heating shows no evidence of material flowing down due to gravity, which could cause the sample to be wider at the bottom or to generate another, flatter, wavy shaped surface feature with a wavelength similar to the size of the laser spot. The driven force to flatten the uneven surface feature is surface tension, which tends to reduce the high surface energy from the acute angle (Figure 4b) between layers. Compared with the other literature that uses a chemical post-process and laser post-process [34,36], the improvement of this work in the surface roughness is the most significant.

### 3.2. Effect of Laser on Chemical Structure (FTIR)

Although this technique can improve the surface finish to a large extent, it remains unknown whether the polymer degradation induced by the high temperature would cause any detrimental effect. To understand the possible chemical structure alteration from laser local heating, FTIR was used to characterize the surfaces of four samples.

**Figure 6:**
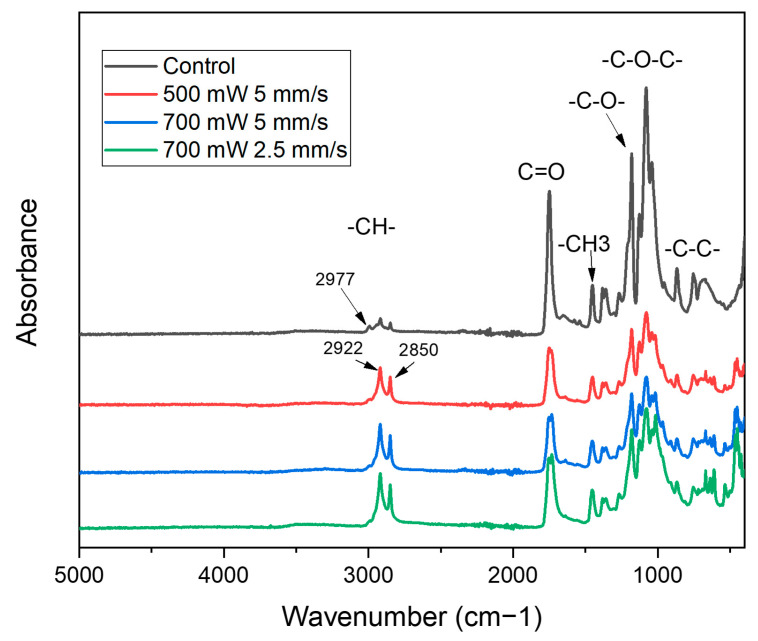
FTIR absorbance data for four samples. To more clearly identify all the peaks, a 0.1 gap is added between each curve.

Shown in Figure 6 is the FTIR result of the control (no laser) and three laser samples that were observed to have a decent surface finish. The main difference between the control and laser-heated samples is the peak at 2922 and 2850 cm^−1^, as well as the overall height of the original PLA peaks between 2000 and 400 cm^−1^.

When analyzing the C-H stretching modes in the 2950–2800 cm^−1^ region [43], it is observed that the relative intensity of the 2922 cm^−1^ increases as the laser power increases compared with the nearby bands in the same spectrum. This effect is also seen, though to a lesser extent, for the 2850 cm^−1^ component. This suggests that these peaks are already present, but weaker, in the PLA spectrum of the control sample. The addition of laser-induced degradation changes their relative intensities, leading to an increased relative absorption in this region. The relative intensity at 2997 cm^−1^ (asym.) also slightly decreases in the 700 mW samples compared with other bands in the same region (i.e., 2922 and 2850 cm^−1^), while the intensity at 2922 and 2850 cm^−1^ increases with the laser power (2.5 mm/s print speed represents double laser power when compared with 5 mm/s). This could be due to a decrease in the polylactic component, which is the main contributor to the C-H stretching bands in the composite spectra. The literature also suggests the same observation in the 2950–2800 cm^−1^ region [43,44].

The reduction in the peak intensity between 2000 and 400 cm^−1^ for the surface of the laser-treated samples further supports the decrease in the PLA component. The peak intensities for PLA in this region are slightly higher for the laser-treated sample at 700 mW and 2.5 mm/s when compared with the two 5 mm/s samples. This indicates an increase in the PLA component with the decrease in print speed (representing a higher laser power), while the peak intensities at 2922 and 2850 cm^−1^ are slightly higher, which should represent a larger amount of degradation. The authors presume the reason for this to be the result of the surface roughness difference that affected the FTIR measurement due to the contact-based diamond ATR characterization method. Therefore, the slightly rougher surface in the 5 mm/s group results in a lower-intensity absorbance.

### 3.3. Mechanical Strength and Fracture Behavior

Polymer degradation usually leads to weak mechanical strength. Even though a smooth surface finish was achieved with this technology, degradation was observed. To better understand if the printed part with a smooth surface can be used as a normal part, the affected region was further investigated from the perspectives of depth and mechanical strength to explore the impact of the laser.

**Figure 7:**
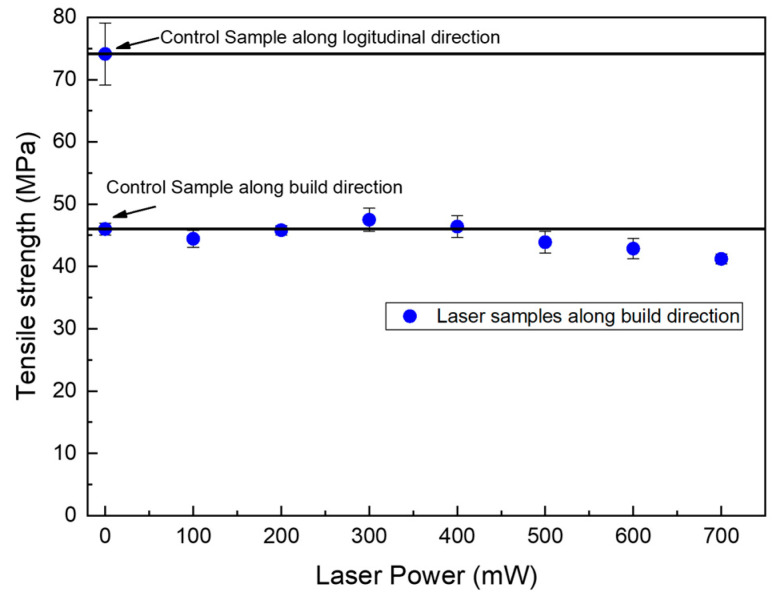
Tensile strength of laser-treated samples printed at 5 mm/s.

As shown in Figure 7, tensile strength was measured in this work. Fracturing was carried out on the interlayer interface of each sample. There is no obvious rise or decline in the tensile strength, despite a minor decrease at 700 mW, which is probably the result of polymer degradation [40]. Hence, the mechanical strength is considered to receive no impact from the laser surface heating. Note that the sample for the tensile test is a single wall sample; any deposited material for the inner walls of multi-wall samples and infills are not affected by the laser.

**Figure 8:**
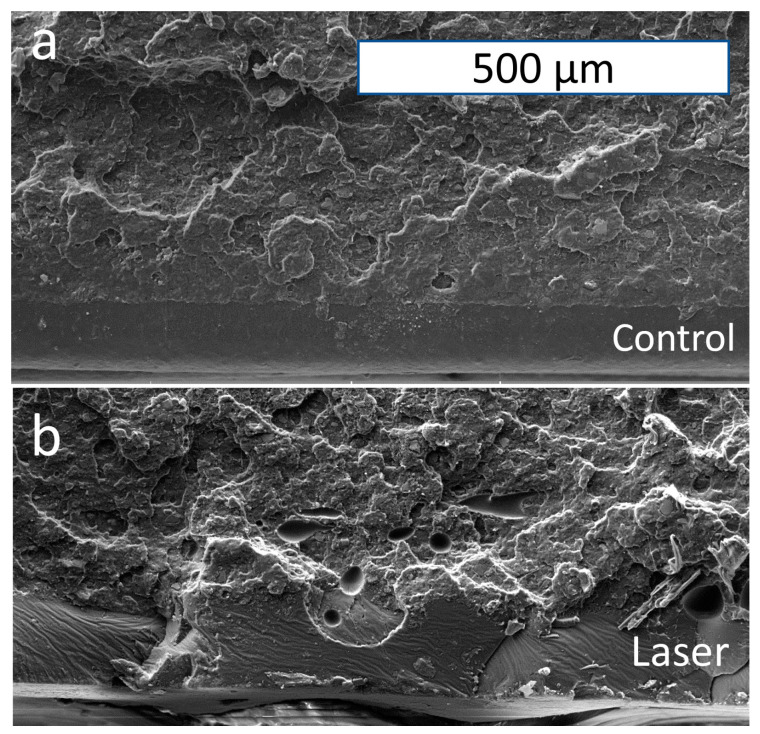
SEM image of fracture surface: (**a**) control sample; (**b**) laser sample 5 mm/s 700 mW. (The same scale bar is used.).

The fracture surface of each sample was imaged with SEM (Figure 8) to explore how this technique exerts its influence in the Y-direction (which is not the building direction, but along the laser beam). In the control sample, there is a smooth area in the lower region with no plastic-fracturing-induced deformation, which represents the round shape surface of the deposited track. There is a similar feature of the inner fracture for the control sample and the sample laser-heated at 700 mW. Nevertheless, near the laser-heated surface, the bottom region is found to show a distinctive fracture feature.

As shown in Figure 8b, the SEM image is observed to have some glossy surface at the bottom. The fracture surface is smoother in the neighboring region than in the upper region. Plastic deformation in a smaller depth but a high volume is observed in the upper region. The layers are inferred to have solid bonding from these plastic deformation regions. Nevertheless, the previously entangled polymer chains are extended and disentangled near the interlayer interface [41,42,45] and are pulled out during the fracture due to the weak interface bonding.

However, under the effect of laser surface heating, the region treated is observed to be smoother in its fracture surface. This means that the gap between the layers is filled due to the surface reflow to a certain degree. Nonetheless, there is not enough time for relaxation [18] or complete reptation [46], as observed from the tensile strength data, to form a region that is isotropic and solid. Surface reflow is driven by surface tension. According to Figure 8a,b, the width of the unbounded region has some similarity with the smoother region’s depth. Hence, laser treatment seeks to increase the surface smoothness by merely affecting the surface material at the side in a small thickness. The deposited track is not subject to the degradation impact of laser heating. From the tensile strength data, a similar conclusion can be drawn that the mechanical strength receives no significant impact from the heating process.

### 3.4. Surface Heating on a Curved Surface

**Figure 9:**
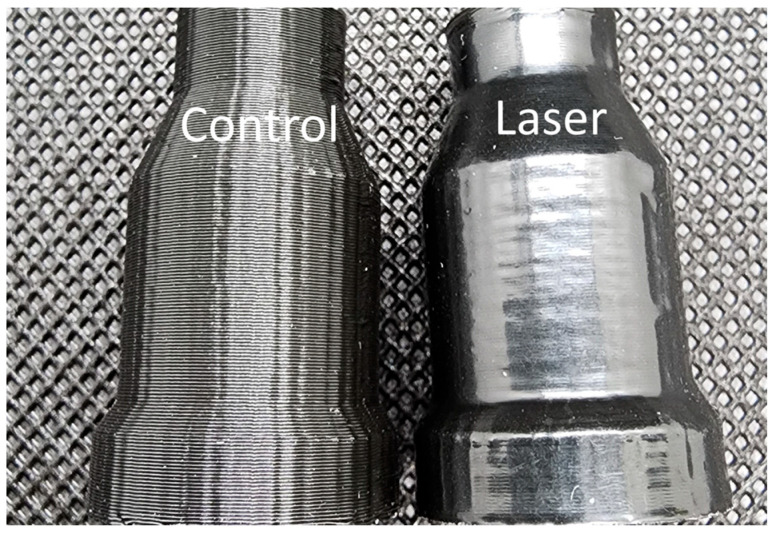
Optical image of hose adapters printed.

A curved surface was involved to examine how the heating process works where two hose adapters were customized and fabricated (Figure 9). The control hose adapter sample (left side of Figure 9) shows a clear, repeated wave-shape feature on the side surface, while the surface of the laser sample is much smoother and even, with light-reflection appearance. The design of this hose adapter sample contains curvature along both the horizontal and the vertical directions. Decent surface finish appearance was observed on both directions with the orbiting laser technique. It can be observed from the light-reflection effect on the control sample that there are some step-shaped features from left to right. This exterior feature is due to the rotation of the orbiting laser that gently affects the form of the deposited track. Another vertical surface feature from this process can be witnessed on the right side of both hose adapter samples. This is due to the inconsistency of the material deposition due to the nozzle lifting at layer changes; however, this defect can be reduced by optimizing retraction at the layer change in the slicing software. This process considerably improved the surface finish for the curved sample.

## 4. Conclusions

With the focus on polymer material extrusion-based 3D printing, this work seeks to explore the role of laser surface heating, applied in the process of printing, in improving the surface finish of printed items. Tests were conducted to measure the mechanical strength and surface roughness under the influence of laser heating. The value of surface roughness (Ra) was found to have an obvious decline from 15 to 2 microns. Surface heating was observed to have no impact on mechanical strength. A significant increase in surface smoothness was achieved using this technique, even when the surface is curved. The technique also has the potential to be implemented on commercial material extrusion-based polymer 3D printers. The design of the technique also delivers ideas to improve other additive manufacturing techniques, such as the in-process fast-curing technique with secondary energy input for house 3D printing and in-process defect characterization with X-ray scattering for direct energy deposition. The commercialization of this technique will benefit the FFF 3D-printer market and workforce with solutions for build quality.

## Data Availability

Not applicable.
